# Understanding Preeclampsia: Integrating Placental Dysfunction, Immune Dysregulation and microRNA-Mediated Epigenetic Regulation

**DOI:** 10.3390/ijms27104281

**Published:** 2026-05-11

**Authors:** Lucia Maria Procopciuc, Gabriela Valentina Caracostea, Adriana Corina Hangan, Roxana Liana Lucaciu

**Affiliations:** 1 Department of Medical Biochemistry, Faculty of Medicine, “Iuliu-Hațieganu” University of Medicine and Pharmacy, 400349 Cluj-Napoca, Romania; lprocopciuc@umfcluj.ro; 2 Medicover Hospital, Suceagu, 407062 Cluj, Romania; caracostea1@yahoo.com; 3 Department of Inorganic Chemistry, Faculty of Pharmacy, “Iuliu-Hațieganu” University of Medicine and Pharmacy, 400012 Cluj-Napoca, Romania; 4 Department of Pharmaceutical Biochemistry and Clinical Laboratory, Faculty of Pharmacy, “Iuliu-Hațieganu” University of Medicine and Pharmacy, 400349 Cluj-Napoca, Romania

**Keywords:** preeclampsia, placental dysfunction, immune dysregulation, angiogenic imbalance, microRNAs

## Abstract

Preeclampsia is a pregnancy-specific multisystem disorder and a major cause of maternal and perinatal morbidity and mortality worldwide. This narrative review summarizes current evidence on the principal risk factors and pathophysiological mechanisms involved in its development. The disease is best explained by the two-stage model: in stage 1, inadequate trophoblast invasion and incomplete spiral artery remodeling lead to placental hypoperfusion, hypoxia, and oxidative stress; in stage 2, the hypoxic placenta releases anti-angiogenic and pro-inflammatory factors, including soluble fms-like tyrosine kinase-1 (sFlt-1) and soluble endoglin (sEng), which trigger systemic endothelial dysfunction and the maternal clinical syndrome. The review highlights the central role of angiogenic imbalance, immune dysregulation, and chronic inflammation in disease progression. Particular emphasis is placed on maternal risk factors such as primiparity, advanced maternal age, obesity, diabetes mellitus, chronic hypertension, multiple pregnancy, prior preeclampsia, genetic susceptibility, and epigenetic regulation. We also emphasize the contribution of microRNAs in relation to placental hypoxia, trophoblast invasion, angiogenesis, endothelial injury and microchimerism to the development of preeclampsia. The review also examines the role of T helper 1 (Th1)/Th2/Th17/regulatory T cells (Treg) imbalance and uterine natural killer cell dysfunction at the maternal–fetal interface. Improved understanding of these interconnected mechanisms may support earlier diagnosis, better risk stratification, and the development of targeted preventive and therapeutic strategies.

## 1. Introduction

Preeclampsia is a pregnancy-specific multisystem disorder and one of the major hypertensive disorders of pregnancy [1,2,3]. Globally, preeclampsia affects approximately 4–10% of pregnancies and remains a leading cause of maternal and perinatal morbidity and mortality [2]. The condition is responsible for more than 500,000 fetal and neonatal deaths and about 70,000 maternal deaths worldwide each year [4]. It is characterized by the development of new-onset hypertension (systolic blood pressure ≥140 mmHg and/or diastolic blood pressure ≥90 mmHg) after 20 weeks of gestation accompanied by proteinuria (>300 mg in 24 h) and/or evidence of maternal organ dysfunction or uteroplacental dysfunction [5]. According to the criteria established by the International Society for the Study of Hypertension in Pregnancy (ISSHP), maternal organ dysfunction may include acute kidney injury, liver involvement, neurological complications, or hematological abnormalities [6,7].

The only definitive treatment for preeclampsia is the delivery of the placenta and fetus, which is frequently associated with increased neonatal morbidity and mortality related to prematurity. Early-onset preeclampsia may progress rapidly to multi-organ dysfunction, involving several organ systems.

Clinical manifestations can include neurological complications such as headache, seizures, or stroke; hematologic abnormalities including thrombocytopenia and disseminated intravascular coagulation; and acute renal failure and hepatic complications, such as subcapsular hematoma or liver rupture. In severe cases, it may also present as HELLP syndrome, characterized by hemolysis, elevated liver enzymes, and low platelet count [8]. Preeclampsia, if left untreated, can lead to serious complications for both mother and baby [9,10]. These complications can range from short-term issues to long-term health problems. Prompt medical intervention is crucial to minimize these risks. Women who experience preeclampsia have nearly double the lifetime risk of developing cardiovascular diseases, including coronary artery disease, heart failure, stroke, and cardiovascular mortality, compared with women who have uncomplicated pregnancies. This increased risk may become evident within the first 1–3 years after pregnancy and can persist for more than ten years [11]. Fetal complications of preeclampsia include preterm birth, fetal growth restriction, placental abruption [12,13].

Although the clinical manifestations of preeclampsia are well characterized, the underlying pathophysiological mechanisms remain complex and multifactorial. Current evidence supports a multifactorial origin involving abnormal placentation, maternal cardiovascular maladaptation, immune dysregulation, genetic predisposition, and metabolic disturbances. These factors interact through several pathophysiological pathways, including oxidative stress, systemic inflammation, and endothelial dysfunction [12,14]. This endothelial dysfunction is responsible for the clinical manifestations of preeclampsia, including hypertension, proteinuria, coagulation abnormalities, and multi-organ involvement [12,15].

A wide range of maternal, genetic, and environmental risk factors have been associated with the development of preeclampsia, primarily through their effects on placentation, angiogenesis, inflammation, and vascular homeostasis. Risk factors such as obesity, diabetes mellitus, chronic hypertension, genetic and epigenetic susceptibility may exacerbate endothelial dysfunction, inflammatory responses, and metabolic disturbances [16,17].

The purpose of this review is to summarize the current evidence regarding the major risk factors and pathophysiological mechanisms involved in preeclampsia. In contrast to traditional linear models of preeclampsia pathogenesis, which describe a sequential progression from abnormal placentation to endothelial dysfunction, this review proposes a more integrative and dynamic framework. Specifically, it emphasizes the bidirectional interactions between placental hypoxia, maternal immune activation, and epigenetic regulation, particularly microRNA-mediated mechanisms.

## 2. Pathophysiology of Preeclampsia

Normal placental development requires the invasion of trophoblast cells into the maternal decidua and the transformation of spiral arteries into high-capacity vessels. This remodeling process ensures adequate blood flow to the placenta and the developing fetus [18].

The pathogenesis of preeclampsia is complex and multifactorial. The two-stage model of preeclampsia, which includes abnormal placentation, endothelial dysfunction, and angiogenic imbalance, is the most well-known conceptual framework for understanding the disease.

Stage 1 of preeclampsia begins with incomplete trophoblast invasion; the spiral arteries remain narrow and resistant to blood flow. Impaired placental perfusion, which is commonly associated with abnormal placentation and insufficient remodeling of the maternal spiral arteries occurred and uteroplacental perfusion becomes compromised. Although these placental abnormalities play a central role, maternal factors also contribute substantially to the systemic pathophysiological processes involved in the disease [19,20,21]. This early phase generally occurs during the first trimester and corresponds to the differentiation and invasion of extravillous trophoblasts (EVTs) into the decidua, a process essential for establishing adequate uteroplacental circulation and ensuring proper fetal blood supply. The process may start as early as before the 8th week of gestation, while the formation of the uteroplacental circulation is typically completed by approximately the 12th week. Therefore, stage 1 is considered to take place during early pregnancy, roughly before 12–20 weeks of gestation [22].

The differentiation and invasive behavior of EVTs are regulated by numerous molecular signals, including cytokines, growth factors, chemokines, and cell adhesion molecules, which may serve as potential markers of this early stage [19,20]. When trophoblast invasion is inadequate and the physiological transformation of the maternal uterine vasculature is incomplete, maternal uterine blood flow becomes reduced. Placental hypoxia induces oxidative stress and promotes the generation of reactive oxygen species (ROS) within the placenta which may be identified through uterine artery Doppler assessment.

Elevated vascular resistance during early pregnancy reflects impaired placental perfusion, and placental endothelial cells from pregnancies with high-resistance uterine arteries appear to be more vulnerable to tumor necrosis factor-α (TNF-α)-induced injury and apoptosis. In contrast, during normal placental development, the resistance of the uterine arteries progressively declines as trophoblast invasion advances, with major changes occurring during the first trimester [23]. Hypoxic conditions may disrupt the balance of antioxidant enzyme activity within placental tissues. However, placental histopathological findings at this stage are often nonspecific and are not unique to preeclampsia, as similar alterations may also arise from other microscopic injuries or exposure to toxins [24].

**Figure 1:**
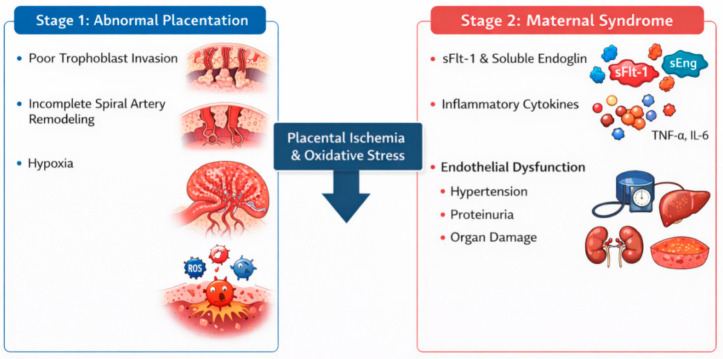
Two-stage model of preeclampsia. Stage 1: Abnormal placentation through poor trophoblast invasion; incomplete spiral artery remodeling and hypoxia determine placental ischemia and oxidative stress; Stage 2: Placental ischemia determines release of sFlt-1, sEng, inflammatory cytokines (TNF-alfa, IL-6) which promote endothelial dysfunction with hypertension, proteinuria, organ damage.

Stage 2 represents the phase in which reduced uteroplacental perfusion interacts with maternal constitutional factors, leading to widespread pathophysiological alterations affecting multiple organ systems and reflecting inadequate blood supply. The clinical manifestations observed in the mother are largely driven by systemic endothelial dysfunction and vascular injury, which have been consistently demonstrated in women with preeclampsia [19]. Placental hypoxia stimulates the release of inflammatory mediators and anti-angiogenic factors into the maternal circulation. These placental signals represent the initiating events in the development of the maternal syndrome [1,15]. The two-stage model of preeclampsia is presented in Figure 1.

One of the most important mechanisms involved in preeclampsia is the imbalance between angiogenic and anti-angiogenic factors. The principal angiogenic and anti-angiogenic factors release by the placenta include vascular endothelial growth factor (VEGF), placental growth factor (PlGF), sFlt-1 and sEng.

sFlt-1 binds and neutralizes pro-angiogenic factors such as VEGF and PlGF and prevents their interaction with endothelial receptors [12,25,26]. The reduction in these angiogenic mediators contributes to endothelial dysfunction and impaired vascular repair, vasoconstriction, increased vascular permeability, and proteinuria [25]. Angiogenic imbalance also reduces nitric oxide (NO) production and increases vasoconstrictors such as endothelin-1 and thromboxane, contributing to the hypertension characteristic of the disease [15].

**Figure 2:**
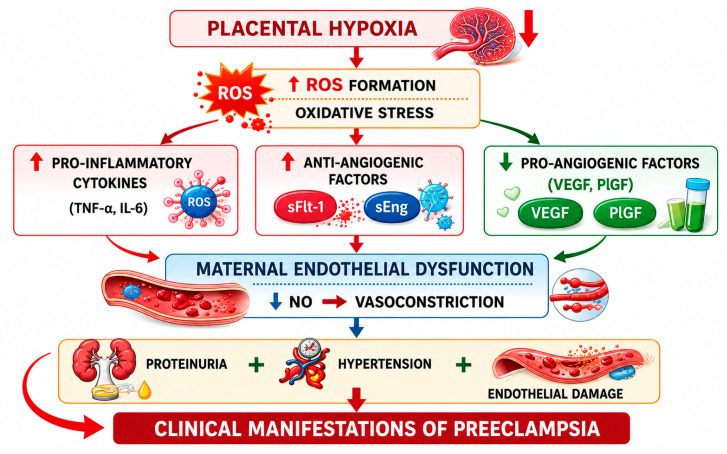
Role of placental hypoxia and oxidative stress in mediation of sFlt-1/PIGF levels. Placental hypoxia determines increased ROS formation and oxidative stress. Oxidative stress determines increased pro-inflammatory cytokines (TNF-α, IL-6) and anti-angiogenic factors (sFlt-1, sEng) and decreased pro-angiogenic factors (VEGF, PlGF). Maternal endothelial dysfunction, vasoconstriction, proteinuria and hypertension have occurred.

Clinical studies have shown that increased sFlt-1 levels and decreased PlGF concentrations can be detected weeks before clinical symptoms appear. Consequently, the sFlt-1/PlGF ratio is currently used as a biomarker for predicting and diagnosing preeclampsia [25]. The role of placental hypoxia in sFlt-1/PIGF balance is presented in Figure 2.

## 3. Maternal Risk Factors for Preeclampsia

### 3.1. Primiparity

Primiparity is a well-established epidemiological risk factor for preeclampsia, with first pregnancies demonstrating a significantly higher incidence compared with multiparous pregnancies [13,27]. The risk has been estimated to be two to three times higher in primiparous women compared with multiparous women [28]. Furthermore, studies have shown that the risk decreases in subsequent pregnancies with the same partner, but increases again when the partner changes, further supporting the role of paternal antigen exposure and immune priming [29].

The most widely accepted explanation is the immunological hypothesis. The mechanisms underlying this association involve maternal immune maladaptation, impaired placentation, systemic inflammation, and incomplete cardiovascular adaptation during the first gestation [30,31].

One of the most widely accepted explanations for the increased risk of preeclampsia in primiparous women is insufficient maternal immune tolerance to paternal antigens expressed by the fetus and placenta. During pregnancy, fetal trophoblast cells express paternal antigens that interact with maternal immune cells at the maternal–fetal interface. In primigravid pregnancies, the maternal immune system encounters these antigens for the first time, which may result in incomplete development of immune tolerance mechanisms, including insufficient expansion of Tregs and an imbalance in T helper cell subsets (Th1/Th2/Th17/Treg) [32,33,34].

This imbalance favors pro-inflammatory Th1 and Th17 responses, increasing the production of cytokines such as TNF-α, interferon gamma (IFN-γ), interleukin-6 (IL-6), and IL-17, which contribute to placental inflammation and endothelial activation [10].

Epidemiological observations further support this immunological hypothesis. Studies have demonstrated that shorter duration of sexual cohabitation before conception, which limits maternal exposure to paternal antigens, is associated with an increased risk of preeclampsia [35,36].

Successful placentation requires deep invasion of EVTs into the decidua and adequate remodeling of spiral arteries into low-resistance vessels. Maternal immune cells, particularly uterine natural killer (uNK) cells, play a central role in regulating these processes through cytokine secretion and receptor interactions with trophoblast human leukocyte antigen (HLA) molecules. Disruption of these interactions may impair trophoblast invasion and spiral artery remodeling [30,31].

In primiparous pregnancies, insufficient immunological adaptation may lead to shallow trophoblast invasion and incomplete spiral artery remodeling, resulting in placental hypoxia and oxidative stress, key mechanisms in the development of preeclampsia [37,38].

Preeclampsia is characterized by exaggerated systemic inflammation and endothelial dysfunction. Placental hypoperfusion caused by abnormal placentation triggers the release of inflammatory cytokines and anti-angiogenic factors into the maternal circulation. These mediators contribute to endothelial injury, hypertension, and multi-organ dysfunction, which represent the clinical manifestations of the disease [10,30]. Recent studies also highlight the role of circulating immune dysregulation and endothelial activation pathways in amplifying the inflammatory cascade associated with preeclampsia [39].

Another explanation for the higher risk in primiparous women involves maternal cardiovascular adaptation. During the first pregnancy, the maternal cardiovascular system undergoes substantial physiological changes, including increased cardiac output, vascular remodeling, and uterine artery adaptation. Multiparous women may benefit from persistent vascular and uterine remodeling induced by previous pregnancies, which can improve placental perfusion and reduce susceptibility to placental malperfusion disorders [31,40].

Overall, these findings support the concept that first pregnancy represents a unique immunological and vascular challenge, during which incomplete maternal adaptation to the fetal–placental unit may predispose to the development of preeclampsia.

### 3.2. Advanced Maternal Age

Advanced maternal age (≥35 years) is an established risk factor for preeclampsia [14]. Several mechanisms have been proposed to explain this association, including vascular aging, impaired placentation, increased oxidative stress, immune dysregulation, and a higher prevalence of maternal comorbidities [8,30,31]. Age-related vascular changes lead to endothelial dysfunction, increased arterial stiffness, and reduced NO availability, which may impair the maternal cardiovascular adaptation required during pregnancy and compromise uteroplacental perfusion [40]. In addition, advanced maternal age has been associated with impaired trophoblast invasion and abnormal spiral artery remodeling, resulting in placental hypoperfusion and ischemia—key events in the pathogenesis of preeclampsia [13]. Imbalances between angiogenic and anti-angiogenic factors may further contribute to disease development [41,42]. Together, these mechanisms facilitate the development of systemic endothelial dysfunction, a key feature in the pathogenesis of preeclampsia.

Maternal aging is also associated with increased oxidative stress and chronic low-grade inflammation, which may further contribute to endothelial injury and placental dysfunction [43,44,45,46]. Furthermore, age-related alterations in immune regulation, including imbalance between pro-inflammatory Th and Tregs cells, may impair maternal–fetal immune tolerance and promote placental inflammation [32].

Finally, older pregnant women have a higher prevalence of underlying conditions such as chronic hypertension, obesity, and metabolic disorders, which further increase the risk of developing preeclampsia [25].

### 3.3. Obesity

Obesity represents one of the most important modifiable risk factors for preeclampsia. Women with a body mass index (BMI) >30 kg/m^2^ have a two- to threefold increased risk of developing the condition [8,30,47,48,49].

Several mechanisms have been proposed to explain this association, including metabolic dysfunction, chronic inflammation, endothelial dysfunction, oxidative stress, altered angiogenesis and impaired placentation [50,51].

One of the key mechanisms is insulin resistance and metabolic dysregulation, which are common in obesity. Elevated circulating lipids, glucose, and insulin levels can impair endothelial function and promote vascular inflammation, increasing susceptibility to hypertensive disorders during pregnancy [48,52].

Obesity is also associated with chronic low-grade inflammation, characterized by increased levels of inflammatory cytokines such as TNF-α, IL-6, and C-reactive protein (CRP). These inflammatory mediators contribute to endothelial activation and may amplify the systemic inflammatory response observed in preeclampsia [50,51,53].

Another important mechanism involves adipokine dysregulation. Adipose tissue in obese individuals produces increased levels of leptin and other pro-inflammatory adipokines, while protective adipokines such as adiponectin are reduced. These changes promote oxidative stress and vascular dysfunction, both of which are central features of preeclampsia [48].

Obesity is associated with increased production of ROS, which decrease NO bioavailability and promote vasoconstriction [15].

Maternal obesity may also impair placental development and trophoblast invasion, leading to inadequate remodeling of the spiral arteries and reduced uteroplacental perfusion. Placental hypoxia subsequently stimulates the release of anti-angiogenic factors such as sFlt-1 [8,31]. Elevated circulating levels of anti-angiogenic factors such as sFlt-1 may contribute to the development of preeclampsia by neutralizing proangiogenic factors such as VEGF and PlGF [12,42]. sFlt-1 binds VEGF and PlGF, preventing their interaction with endothelial receptors and thereby leading to endothelial dysfunction [12,25].

Overall, these metabolic, inflammatory, and vascular alterations create a maternal environment that predisposes to abnormal placentation and the systemic endothelial dysfunction that defines preeclampsia.

### 3.4. Pre-Existing Conditions: Diabetes Mellitus and Chronic Hypertension

Diabetes mellitus, including both pregestational diabetes and gestational diabetes, is an important risk factor for preeclampsia [47]. Women with diabetes have a significantly higher risk of developing preeclampsia compared with non-diabetic pregnant women [8,30,31,54].

One of the principal mechanisms involves chronic hyperglycemia and insulin resistance, which promote oxidative stress and endothelial dysfunction. Elevated glucose levels increase the production of ROS and reduce NO bioavailability, impairing vascular function and maternal cardiovascular adaptation to pregnancy and activation of the renin–angiotensin system. These alterations contribute to hypertension and impaired uteroplacental perfusion [47,48,55].

Diabetes is also associated with chronic low-grade inflammation, characterized by elevated inflammatory cytokines such as TNF-α, IL-6, and CRP, which can exacerbate endothelial activation and vascular injury [50,53,56]. These inflammatory processes contribute to the systemic endothelial dysfunction that characterizes preeclampsia.

Another important mechanism involves abnormal placental development. Hyperglycemia can impair trophoblast invasion and spiral artery remodeling, leading to placental hypoperfusion and ischemia. Placental stress subsequently promotes the release of anti-angiogenic factors such as sFlt-1 and sEng, which trigger maternal endothelial dysfunction [8,30].

Epidemiological studies further support this association. For example, a large population-based cohort study showed that women with pregestational diabetes had a significantly increased risk of preeclampsia compared with non-diabetic women [57,58]. Similarly, another cohort study demonstrated that gestational diabetes was associated with a higher incidence of hypertensive disorders of pregnancy, including preeclampsia [59].

Overall, metabolic disturbances, vascular dysfunction, inflammation, and abnormal placentation in diabetic pregnancies contribute to an increased susceptibility to preeclampsia.

Chronic hypertension is a well-established risk factor for preeclampsia, and women with pre-existing hypertension have a significantly higher risk of developing superimposed preeclampsia during pregnancy [8,31,60]. Several pathophysiological mechanisms have been proposed to explain this association, including endothelial dysfunction, impaired vascular adaptation, placental malperfusion, and chronic inflammation.

One of the key mechanisms involves pre-existing endothelial dysfunction. Chronic hypertension is associated with vascular damage, reduced NO bioavailability, and increased oxidative stress, which impair the ability of the maternal cardiovascular system to adapt to the hemodynamic changes in pregnancy [15,40]. These alterations contribute to abnormal uteroplacental perfusion and increased vascular resistance.

Chronic hypertension may also interfere with normal placentation. Structural changes in the maternal vasculature can impair trophoblast invasion and spiral artery remodeling, leading to reduced uteroplacental blood flow and placental ischemia [30,31]. Placental hypoxia subsequently stimulates the release of anti-angiogenic factors such as sFlt-1 and sEng, which promote systemic endothelial dysfunction.

Another contributing factor is chronic inflammation and vascular remodeling associated with long-standing hypertension. Increased levels of inflammatory mediators and oxidative stress may further damage the endothelium and amplify the inflammatory response characteristic of preeclampsia [8,30].

Epidemiological evidence also supports this relationship. Population-based studies have shown that women with chronic hypertension have a markedly increased risk of developing superimposed preeclampsia, with reported incidence rates ranging from approximately 20% to 50% depending on the severity of the underlying hypertension [61,62,63].

Overall, the combination of pre-existing vascular dysfunction, impaired placental development, oxidative stress, and inflammation in women with chronic hypertension increases susceptibility to preeclampsia.

### 3.5. Multiple Pregnancies

Multiple pregnancies, including twin and higher-order gestations, are associated with approximately a two- to threefold significantly increased risk of preeclampsia compared with singleton pregnancies [64]. This increased risk is thought to result from greater placental mass, increased production of placental factors, and heightened maternal cardiovascular and inflammatory responses [8,30,31,65].

One important mechanism involves the larger placental mass present in multiple pregnancies. A larger placenta produces higher levels of placental-derived factors, including anti-angiogenic proteins such as sFlt-1 and sEng, which contribute to maternal endothelial dysfunction, a central feature of preeclampsia [8,30,66,67]. This imbalance is characterized by increased anti-angiogenic factors (sFlt-1) and decreased proangiogenic factors (VEGF, PlGF). Such an imbalance is considered a central mechanism in the pathogenesis of preeclampsia [12,25].

Multiple gestations are also associated with increased placental metabolic demand and relative placental hypoxia, which may lead to oxidative stress and the release of inflammatory mediators into the maternal circulation [31]. These processes can amplify systemic inflammation and vascular dysfunction.

Epidemiological studies have consistently demonstrated a higher incidence of preeclampsia in multiple pregnancies. For example, population-based studies have shown that twin pregnancies are associated with approximately a two- to threefold higher risk of preeclampsia compared with singleton pregnancies [68,69].

Another contributing factor is the greater hemodynamic burden on the maternal cardiovascular system in multiple pregnancies. Increased blood volume and cardiac output requirements may exceed maternal cardiovascular adaptive capacity, particularly in women with pre-existing risk factors [63,70,71,72], thereby increasing susceptibility to hypertensive disorders of pregnancy [61,66].

Overall, the combination of increased placental mass, elevated anti-angiogenic factors, placental hypoxia, systemic inflammation, and increased maternal cardiovascular load contributes to the higher risk of preeclampsia observed in multiple pregnancies.

### 3.6. Personal History of Preeclampsia

A history of preeclampsia is one of the strongest predictors of preeclampsia in subsequent pregnancies. Women who previously experienced preeclampsia have a significantly increased risk of recurrence, particularly if the condition occurred early in pregnancy or was severe [63,70,71,72]. Several mechanisms have been proposed to explain this association, including persistent maternal vascular dysfunction, genetic susceptibility, and underlying metabolic or immunological abnormalities.

One important mechanism involves persistent endothelial dysfunction and cardiovascular alterations following a pregnancy complicated by preeclampsia. Studies have shown that women with prior preeclampsia may develop long-term vascular changes, including impaired endothelial function and increased arterial stiffness, which may predispose them to hypertensive disorders in subsequent pregnancies [40,71]. Several studies suggest that women with a history of preeclampsia may exhibit long-lasting vascular dysfunction even after pregnancy, which may predispose them to recurrence in subsequent pregnancies [73].

Genetic and familial factors may also contribute to the recurrence of preeclampsia. Both maternal and fetal genetic predispositions affecting angiogenic pathways, immune regulation, and placental development have been implicated in the disease [30,31].

Another proposed mechanism involves persistent abnormalities in placental development and maternal immune responses. Dysregulation of trophoblast invasion and spiral artery remodeling may recur in subsequent pregnancies, particularly in women with underlying vascular or metabolic disorders [8,30].

Epidemiological evidence supports these mechanisms. Large population-based cohort studies have shown that women with a previous history of preeclampsia have a two- to eight-fold higher risk of recurrence in later pregnancies [70,74].

Overall, the recurrence of preeclampsia is likely the result of persistent maternal cardiovascular dysfunction, genetic predisposition, and recurring abnormalities in placental development.

### 3.7. Genetic Factors

Genetic factors play an important role in susceptibility to preeclampsia. Family and twin studies have demonstrated that the risk is increased among women with a family history of preeclampsia.

Evidence from familial aggregation and population-based studies suggests that both maternal and fetal genetic variants contribute to the development of the disease [8,29,30,75]. Recent genomic studies, including genome-wide association studies (GWAS), have identified multiple loci associated with preeclampsia, affecting pathways involved in angiogenesis, immune regulation, placental development, and vascular function [8,30,31,76].

One of the most important mechanisms involves genes regulating angiogenic pathways. Variants affecting VEGF and PlGF signaling may impair placental vascular development and endothelial function. Another consistently identified susceptibility gene is FLT1, which encodes the receptor for VEGF. Placental overexpression of sFlt-1 inhibits VEGF and PlGF, leading to endothelial dysfunction. Increased expression of anti-angiogenic factors such as FLT1, which encodes sFlt-1, can inhibit VEGF and PlGF signaling and contribute to endothelial dysfunction, a central feature of preeclampsia. One variant 936C/T in the VEGF gene has been described as a modulating factor in preeclampsia, which affects the angiogenic balance in preeclamptic mothers, as well as their pregnancy outcome [8,30,67,77].

Genome-wide association studies have shown that variants near the FLT1 locus are significantly associated with an increased risk of preeclampsia [76,78].

Genes involved in immune regulation at the maternal–fetal interface are also important. For example, variants in Endoplasmic Reticulum Aminopeptidase 1 (ERAP1) and Endoplasmic Reticulum Aminopeptidase 2 (ERAP2), which regulate antigen processing and presentation in immune pathways, have been associated with preeclampsia and eclampsia in genetic association studies. ERAP1 plays a key role in the processing of peptides that will be presented by Major Histocompatibility Complex (MHC) class I molecules (also called HLA class I in humans) [79].

ERAP1 is involved in antigen processing and presentation, regulation of immune responses and inflammatory pathways. Because antigen presentation influences how the maternal immune system responds to fetal tissues, variations in ERAP1 activity may affect preeclampsia. ERAP1 and ERAP2 have attracted attention in reproductive immunology because antigen presentation is crucial for maternal–fetal immune tolerance. The maternal immune system must tolerate fetal tissues expressing paternal antigens while maintaining the ability to respond to infections. Variations in ERAP1 and ERAP2 activity may influence the peptide repertoire presented by HLA molecules at the maternal–fetal interface, particularly in trophoblast cells interacting with maternal uNK cells [80,81].

STOX1 (Storkhead Box 1) is a transcription factor gene that plays an important role in placental development and regulation of trophoblast function and oxidative stress responses. It has been widely studied because of its possible involvement in preeclampsia and other pregnancy-related disorder. Because trophoblast cells are responsible for implantation and placental invasion into the maternal uterine tissue, proper STOX1 activity is essential for normal placental function [82]. Certain STOX1 polymorphisms have been associated with early-onset preeclampsia [83].

Another group of susceptibility genes involves immune regulation at the maternal–fetal interface. Interactions between maternal killer cell immunoglobulin-like receptor (KIR) genes expressed on uterine natural killer cells and fetal HLA-C genes expressed on trophoblasts influence trophoblast invasion and spiral artery remodeling. Certain maternal KIR haplotypes combined with specific fetal HLA-C variants have been associated with an increased risk of preeclampsia [84].

The NOS3 gene, also known as endothelial nitric oxide synthase (eNOS), encodes endothelial NO synthase, an enzyme responsible for the production of NO in vascular endothelial cells. NO plays a critical role in maintaining vascular homeostasis by promoting vasodilation, inhibiting platelet aggregation, and regulating vascular tone. Genes involved in endothelial function and vascular regulation may contribute significantly to susceptibility to preeclampsia. In normal pregnancy, NO plays a key role in maintaining vascular tone, regulating blood flow, and promoting proper placental perfusion. Polymorphisms in the NOS3 gene have been associated with reduced NO bioavailability, endothelial dysfunction, and impaired vascular adaptation, all of which are characteristic features of preeclampsia. However, several polymorphisms in the NOS3 gene, including Glu298Asp (rs1799983) and T-786C, have been associated with reduced enzyme activity and decreased NO bioavailability. This reduction may contribute to endothelial dysfunction and impaired placental perfusion, key features in the pathogenesis of preeclampsia [33,85].

Additionally, genes involved in immune and inflammatory responses, such as TNF-α and IL-10, may influence inflammatory pathways and immune tolerance during pregnancy, thereby affecting susceptibility to preeclampsia [39]. Polymorphisms in the TNF gene promoter region, particularly the TNF-α −308 G/A polymorphism, have been associated with increased cytokine production and may contribute to genetic susceptibility to preeclampsia in certain populations. In contrast to TNF-α, IL-10 is an anti-inflammatory cytokine that plays a critical role in maintaining maternal–fetal immune tolerance. IL-10 suppresses excessive inflammatory responses by inhibiting the production of pro-inflammatory cytokines such as TNF-α, IL-1β, and IL-6. During normal pregnancy, IL-10 contributes to regulation of immune tolerance at the maternal–fetal interface, protection of trophoblast cells from immune-mediated damage. Reduced IL-10 expression or functional polymorphisms in the IL10 gene promoter region (such as −1082A/G, −819C/T, and −592C/A variants) have been associated with increased inflammatory responses and may predispose individuals to preeclampsia [86,87].

Genetic variations in genes involved in the renin–angiotensin–aldosterone system (RAAS) have been implicated in the pathogenesis of preeclampsia. RAAS plays a central role in blood pressure regulation, vascular tone, and fluid balance [88]. Human hypertension has been recognized as one of the most important risk factors for the development of cardiovascular diseases such as coronary heart disease (CHD), myocardial infarction (MI) or stroke, all of which are principal causes of human cardiovascular morbidity and mortality [1,2]. Alterations in this pathway may contribute to abnormal maternal cardiovascular adaptation during pregnancy [89]. Several polymorphisms in RAAS-related genes have been associated with increased susceptibility to preeclampsia. For example, variants in the angiotensinogen gene (AGT), particularly the M235T or T704C polymorphisms, have been linked to increased plasma angiotensinogen levels and enhanced angiotensin II production, which can promote vasoconstriction and endothelial dysfunction [89,90]. Similarly, the angiotensin-converting enzyme (ACE) insertion/deletion (I/D) polymorphism has been associated with increased ACE activity and elevated angiotensin II levels, contributing to vascular dysfunction and hypertension in pregnancy [91]. Polymorphisms in the angiotensin II type 1 receptor gene (AGTR1), such as A1166C, may also influence susceptibility to preeclampsia by increasing receptor expression and enhancing the vasoconstrictive and pro-inflammatory effects of angiotensin II [89,92].

These genetic variants may lead to increased vascular resistance, impaired placental perfusion, and abnormal spiral artery remodeling, which are key mechanisms involved in the development of preeclampsia. Overall, alterations in RAAS genes—including AGT, ACE, and AGTR1—may contribute to the pathogenesis of preeclampsia by promoting vasoconstriction, endothelial dysfunction, and placental hypoperfusion. Angiotensin II type 1 receptors (AT1-AAs) are capable of crossing the placenta and entering the fetal circulation. By activating AT1 receptors, AT1-AAs can alter intracellular calcium signaling and trigger downstream pathways that contribute to the development of preeclampsia [93,94]. AT1-AAs also could increase sFlt-1 and sEng and thus cause damage to the endothelium and target organs [95].

**Figure 3:**
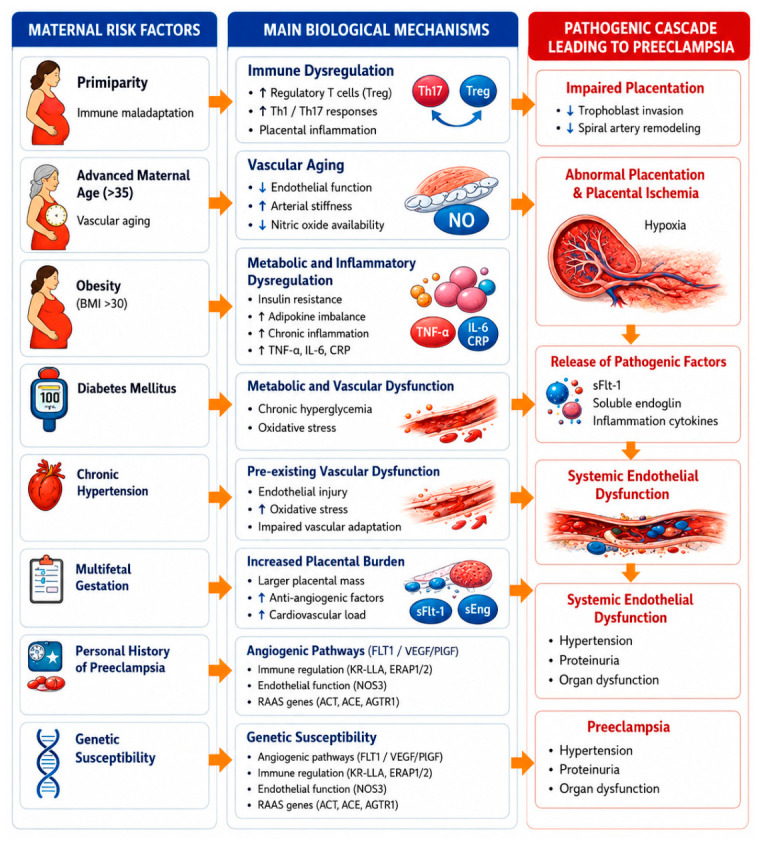
Maternal risk factors and pathophysiological mechanisms contributing to the development of preeclampsia. Maternal risk factors—including primiparity, advanced maternal age, obesity, diabetes mellitus, chronic hypertension, multifetal gestation, prior history of preeclampsia, and genetic susceptibility—promote biological alterations such as immune dysregulation, endothelial dysfunction, metabolic and inflammatory imbalance, and vascular abnormalities. These processes impair trophoblast invasion and spiral artery remodeling, leading to abnormal placentation and placental ischemia. The ischemic placenta releases anti-angiogenic and inflammatory factors (e.g., sFlt-1 and sEng) into the maternal circulation, resulting in systemic endothelial dysfunction and the clinical manifestations of preeclampsia, including hypertension, proteinuria, and organ dysfunction.

Maternal risk factors and their risk on preeclampsia development are presented in Figure 3.

## 4. Epigenetic Modifications in Preeclampsia

Increasing evidence suggests that epigenetic regulators, particularly microRNAs (miRNAs), play a crucial role in the pathogenesis of preeclampsia by modulating placental development, angiogenesis, immune responses, and trophoblast invasion [8,96].

MicroRNAs are small non-coding RNA molecules, approximately 20–24 nucleotides in length, that regulate gene expression post-transcriptionally through binding to complementary sequences within messenger RNA (mRNA). This interaction leads to mRNA degradation or inhibition of translation, thereby controlling a wide variety of biological processes, including proliferation, differentiation, apoptosis, and angiogenesis [97,98]. In pregnancy, miRNAs are essential regulators of placental development, trophoblast differentiation, angiogenesis, and immune tolerance.

In preeclampsia, numerous miRNAs are dysregulated in placental tissue and maternal circulation. These molecules influence key pathogenic mechanisms such as trophoblast invasion, spiral artery remodeling, angiogenesis, oxidative stress, and inflammatory signaling pathways. Placental tissues express unique miRNA clusters, including C19MC, C14MC, and miR-371-373, which are involved in regulating trophoblast proliferation and invasion. Dysregulation of these miRNAs has been associated with pregnancy complications, particularly preeclampsia [99]. Importantly, many placental miRNAs are released into maternal circulation through exosomes and microvesicles, making them promising non-invasive biomarkers for early diagnosis and as potential therapeutic targets in preeclampsia [100,101].

Among the most extensively studied miRNAs implicated in preeclampsia are miR-210 and miR-155, which influence key mechanisms such as placental hypoxia responses, angiogenesis, inflammation, and trophoblast invasion.

### 4.1. miR-210 and Placental Hypoxia

One of the most consistently upregulated miRNAs in preeclamptic placentas is miR-210, often referred to as the “hypoxamiR” due to its strong induction under hypoxic conditions [102]. Placental hypoxia represents a central feature of preeclampsia, resulting from inadequate spiral artery remodeling and impaired trophoblast invasion. Hypoxic conditions activate hypoxia-inducible factor-1α (HIF-1α), which subsequently upregulates miR-210 expression [103].

miR-210 affects multiple molecular pathways relevant to preeclampsia, such as mitochondrial metabolism, trophoblast invasion, and angiogenesis. miR-210 targets ISCU (iron–sulfur cluster scaffold protein), a key component of mitochondrial electron transport chains. Inhibition of ISCU leads to mitochondrial dysfunction and increased ROS production [104]. Moreover, miR-210 suppresses genes involved in trophoblast migration and invasion, including EFNA3 (Ephrin A3), homeobox A9 (HOXA9), and potassium channel modulatory factor 1 (KCMF1). This inhibition contributes to defective trophoblast invasion, a hallmark of preeclampsia [105]. miR-210 negatively regulates angiogenic pathways by targeting genes involved in vascular development, contributing to placental vascular dysfunction. Studies have demonstrated that miR-210 is significantly upregulated in both placental tissue and maternal plasma in women with preeclampsia. Consequently, overexpression of miR-210 promotes oxidative stress, impaired trophoblast invasion, and placental vascular abnormalities, all central features in the development of preeclampsia [105,106].

### 4.2. miR-155 and Inflammatory Pathways

Another miRNA strongly implicated in the pathophysiology of preeclampsia is miR-155, which plays a critical role in immune regulation and inflammation. miR-155 expression is significantly elevated in preeclamptic placental tissues and maternal circulation, suggesting that it contributes to the systemic inflammatory response observed in preeclampsia [107,108].

One of the key targets of miR-155 is eNOS. By suppressing eNOS expression, miR-155 reduces NO production, which leads to vasoconstriction, endothelial dysfunction, and increased vascular resistance. These effects contribute directly to maternal hypertension, a defining feature of preeclampsia [109]. miR-155 also modulates angiogenic signaling pathways by targeting VEGF signaling components. Suppression of these pathways disrupts endothelial cell proliferation and vascular remodeling, further contributing to placental vascular insufficiency [110]. In addition, miR-155 enhances pro-inflammatory cytokine production, including TNF-α, IL-6, and IL-1β. These cytokines exacerbate systemic inflammation and endothelial damage [111].

**Figure 4:**
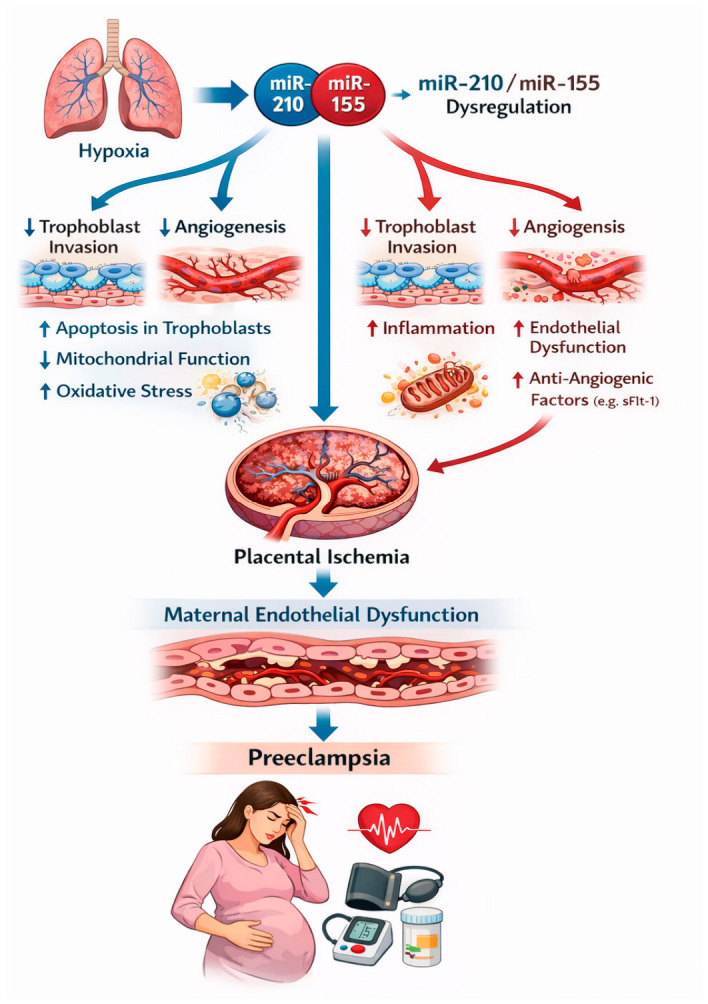
Hypoxia induces dysregulation of miR-210 and miR-155, which disrupt multiple placental processes. miR-210 impairs trophoblast invasion, reduces angiogenesis, increases apoptosis, decreases mitochondrial function, and elevates oxidative stress. miR-155 reduces trophoblast invasion and angiogenesis, promotes inflammation, endothelial dysfunction, and increases anti-angiogenic factors (e.g., sFlt-1). These alterations lead to placental ischemia and maternal endothelial dysfunction, ultimately contributing to the development of preeclampsia.

The role of miR-155 and miR-210 in preeclampsia development is presented in Figure 4.

### 4.3. Chromosome 19 microRNA Cluster (C19MC)

The chromosome 19 microRNA cluster (C19MC) is one of the largest miRNA clusters in humans and is expressed almost exclusively in the placenta. Its expression can be detected as early as the fifth week of gestation and progressively increases in placental trophoblasts from the first to the third trimester [112,113].

miRNAs derived from the C19MC cluster are secreted by primary human trophoblasts and released into the maternal circulation within exosomal vesicles. These circulating exosomal miRNAs can modulate maternal immune cell function, contributing to the establishment of maternal–fetal immune tolerance and playing an important role in antiviral defense during pregnancy [23,100,114,115].

Different miRNAs within the C19MC cluster, such as miR-519d, miR-515-5p, miR-517-5p, and miR-518b, are essential for proper placental development and regulation of trophoblast migration.

miR-519d regulates the invasive phenotype of EVTs and can suppress trophoblast invasion and migration. miR-515-5p has been found to be upregulated in placentas from pregnancies complicated by preeclampsia [19,116]. Similarly, miR-517-5p is highly expressed in preeclamptic placentas and reduces the proliferative and invasive capacity of cells from human choriocarcinoma cell lines. Upregulation of miR-517a/b/c decreases trophoblast invasion and increases the release of the anti-angiogenic factor sFlt-1 [116]. In addition, abnormal overexpression of miR-518b in the preeclamptic placenta may promote excessive trophoblast proliferation.

Dysregulation of C19MC expression can disrupt key placental processes, including trophoblast differentiation, invasion, and angiogenesis [112,116].

### 4.4. Regulation of Angiogenesis by miRNAs

Proper placental angiogenesis is essential for maintaining adequate maternal–fetal circulation. In normal pregnancy, the placenta develops an extensive vascular network supported by pro-angiogenic factors such as VEGF and PlGF. In preeclampsia, angiogenesis becomes severely disrupted due to an imbalance between pro-angiogenic and anti-angiogenic factors [117]. Several miRNAs participate in regulating angiogenic pathways in the placenta.

Certain miRNAs are overexpressed in preeclampsia and suppress angiogenic signaling. miR-210 inhibits endothelial cell migration and suppresses VEGF signaling. miR-16 and miR-29 reduce VEGF expression and vascular development [118]. On the other hand, some miRNAs normally promote angiogenesis but become dysregulated in preeclampsia. miR-126 enhances VEGF signaling and supports endothelial integrity. Reduced expression of miR-126 in preeclampsia contributes to impaired vascular development [119].

Experimental studies have demonstrated that altered expression of specific miRNAs, such as miR-302a, can regulate VEGF expression and thereby influence placental angiogenesis [120].

miR-155 regulates trophoblast proliferation, migration, and apoptosis through multiple molecular targets, including components of the PI3K signaling pathway. Overexpression of miR-155 suppresses trophoblast invasion and migration, while inhibition of miR-155 enhances trophoblast viability and invasive capacity. These findings suggest that dysregulated miR-155 expression contributes to shallow trophoblast invasion, a key pathological feature of preeclampsia [121]. Additionally, miR-155 participates in oxidative stress pathways and inflammatory signaling. The miR-155/Nrf2 regulatory axis has been shown to modulate oxidative stress responses in trophoblast cells and contribute to placental inflammation and endothelial dysfunction in preeclampsia [122]. Other miRNAs have also been implicated in angiogenic regulation. For example, miR-224-5p has been shown to modulate trophoblast function and vascular signaling pathways through targeting specific molecular mediators involved in placental development, suggesting a potential protective or regulatory role in preeclampsia [120].

Through these regulatory mechanisms, miRNAs contribute to the imbalance between pro-angiogenic and anti-angiogenic factors, ultimately leading to endothelial dysfunction and the maternal clinical manifestations of preeclampsia, including hypertension and proteinuria [98,105].

### 4.5. miRNAs and Trophoblast Invasion

Successful placentation requires the differentiation of cytotrophoblasts into invasive EVTs to invade maternal decidua and remodel maternal spiral arteries. This process transforms the uteroplacental circulation into a low-resistance system that ensures adequate blood supply to the fetus. In preeclampsia, trophoblast invasion is shallow and incomplete, resulting in reduced placental perfusion [123,124].

MicroRNAs regulate several molecular pathways involved in trophoblast migration, proliferation and invasion. Dysregulated miRNA expression can interfere with signaling pathways controlling cell adhesion, extracellular matrix degradation, and cytoskeletal organization. As mentioned previously, miR-210 suppresses genes involved in cell migration and invasion under hypoxic conditions. Increased expression of miR-210 leads to reduced trophoblast motility and shallow placentation [106]. miR-155 also inhibits trophoblast invasion through regulation of cyclin-dependent kinase inhibitors and inflammatory mediators, impairing trophoblast proliferation and migration [109]. Other miRNAs implicated in trophoblast invasion include miR-34a, which suppresses NOTCH signaling and reduces trophoblast proliferation, and miR-376c, which normally promotes trophoblast invasion and decreased expression in preeclampsia and contributes to shallow placentation [125]. Collectively, dysregulated miRNA expression results in defective trophoblast invasion and inadequate spiral artery remodeling, leading to placental hypoxia and subsequent maternal syndrome.

Dysregulated microRNAs involved in the pathogenesis of preeclampsia is presented in Table 1.

**Table 1:** Dysregulated microRNAs involved in the pathogenesis of preeclampsia: Expression patterns, molecular targets, mechanisms, and biological effects.

miRNA	Expression in Preeclampsia	Main Targets	Mechanism	Biological Effect in Preeclampsia	Reference
miR-210	Upregulated	ISCU, EFNA3, HOXA9, KCMF1	Suppresses mitochondrial metabolism genes, inhibits trophoblast migration and angiogenesis	Mitochondrial dysfunction, increased ROS, impaired trophoblast invasion, placental vascular dysfunction	[105,108,126,127,128]
miR-155	Upregulated	eNOS, VEGF signaling components, PI3K pathway components	Inhibits NO production, suppresses angiogenic signaling, modulates inflammatory cytokines	Vasoconstriction, endothelial dysfunction, reduced trophoblast invasion, systemic inflammation	[114,128,129,130,131,132,133,134]
miR-16	Upregulated	VEGF	Suppresses angiogenic signaling	Impaired vascular development	[114]
miR-29	Upregulated	VEGF	Suppresses angiogenic signaling	Impaired vascular development	[114]
miR-126	Downregulated	VEGF signaling	Normally promotes angiogenesis; reduced expression impairs vascular integrity	Impaired placental vascular development	[134]
miR-302a	Dysregulated	VEGF	Modulates VEGF expression	Alters placental angiogenesis	[128]
miR-224-5p	Dysregulated	Placental vascular mediators	Modulates trophoblast function and vascular signaling	Potential protective/regulatory role in preeclampsia	[128]
miR-34a	Upregulated	NOTCH signaling	Suppresses trophoblast proliferation	Reduced trophoblast proliferation, shallow placentation	[133]
miR-376c	Downregulated	Unknown (promotes trophoblast invasion)	Normally promotes trophoblast invasion; decreased expression impairs invasion	Shallow placentation, defective spiral artery remodeling	[133]

### 4.6. miRNA Associated with Microchimerism in Preeclampsia

Microchimerism refers to the bidirectional trafficking of fetal and maternal cells across the placenta during pregnancy. Fetal cells can enter the maternal circulation and persist in maternal tissues for years after delivery [135].

In normal pregnancy, fetal microchimeric cells may participate in immune tolerance and tissue repair mechanisms. In preeclampsia, placental ischemia and oxidative stress increase the release of trophoblast debris, extracellular vesicles, and fetal cells into the maternal circulation [136,137]. These fetal-derived components activate maternal leukocytes, promote systemic inflammation and contribute to immune activation and endothelial injury, as well as to increased production of pro-inflammatory cytokines, processes which take place in the second stage of preeclampsia [136,138].

Therefore, fetal microchimerism may amplify the maternal inflammatory cascade during stage 2 of preeclampsia and contribute to the systemic features of the disease [20,138].

Several miRNAs have been implicated in mechanisms related to microchimerism in preeclampsia, primarily through their presence in placental extracellular vesicles and exosomes and their capacity to regulate maternal immune responses, endothelial function, and trophoblast activity [139,140]. The C19MC-derived miRNAs enter the maternal circulation through exosomes, where they can interact with maternal immune cells and influence immune tolerance mechanisms during pregnancy [112,114,141,142]. The miR-517-3p, miR-517a/b/c and miR-520 families originate from trophoblast cells and circulate in maternal blood within extracellular vesicles. They may accompany fetal cells entering the maternal circulation and therefore participate in microchimerism-related maternal immune modulation, influencing trophoblast migration and spiral artery remodeling [114,142]. They may contribute to abnormal placentation and impaired maternal immune tolerance. miR-203a-3p has been shown to regulate macrophage inflammatory responses, while miR-146a-5p modulates inflammatory and endothelial signaling pathways [143,144].

miR-210 is released from stressed trophoblasts in EVs. It alters maternal endothelial cells and immune cells, increasing pro-inflammatory cytokines and contributing to the maternal systemic response. The impact is greater because it enhances immune activation associated with fetal cell microchimerism [78,145].

miR-155 has a major role in microchimerism because it modulates nuclear factor kappa B (NF-κB) signaling in maternal leukocytes exposed to fetal cells, increasing the inflammatory response to fetal microchimeric cells. Thus, it supports systemic endothelial dysfunction and immune-mediated damage in preeclampsia [146].

miR-223 is a microRNA enriched in trophoblast-derived extracellular vesicles and plays a key role in mediating communication between the fetus and maternal tissues. In the context of microchimerism, miR-223 influences maternal vascular and immune cells, helping to maintain immune tolerance and support proper vascular adaptation. Downregulation of miR-223 can disrupt angiogenesis and impair maternal immune adaptation, increasing maternal tissue reactivity to microchimeric fetal cells. This dysregulation contributes to endothelial dysfunction and hypertension, central features of preeclampsia. Lu et al. (2022) demonstrated the association of placenta-derived exosomal miRNAs with preeclampsia pathophysiology, while Liu et al. (2024) specifically showed that miR-223-3p downregulation promotes inflammasome-mediated inflammatory responses in preeclamptic placentas [147,148].

miR-148a and miR-152 are important regulators of trophoblast immune signaling and play a key role in shaping maternal immune tolerance at the maternal–fetal interface. These microRNAs modulate the expression of HLA-G, a non-classical major histocompatibility complex molecule expressed by trophoblasts that is critical for suppressing maternal immune responses to fetal antigens. When levels of miR-148a and miR-152 are reduced, HLA-G expression may be dysregulated, leading to enhanced maternal recognition of fetal cells and a breakdown in immune tolerance. This reduction in immune suppression can amplify the maternal inflammatory response to fetal microchimeric cells circulating in the maternal circulation, contributing to the systemic inflammation and immune activation observed in preeclampsia. Munjas et al. (2021) provide evidence that altered placental microRNA profiles, including miR-148a and miR-152, are associated with impaired immunomodulatory pathways in preeclampsia [112].

miR-370-3p, miR-144, miR-21, and members of the miR-29 family are additional placenta-derived microRNAs that have been implicated in the pathogenesis of preeclampsia through their presence in exosomes released by trophoblasts, often alongside fetal microchimeric cells. These miRNAs are trafficked into the maternal circulation within extracellular vesicles and can interact with maternal endothelial and immune cells, as well as influence trophoblast behavior. By modulating key signaling pathways involved in angiogenesis, apoptosis, immune regulation, and extracellular matrix remodeling, dysregulated expression of these miRNAs can exacerbate endothelial dysfunction and inflammatory responses when maternal tissues are exposed to fetal microchimeric cells. For example, altered levels of miR-370-3p have been linked to decreased trophoblast invasion and increased apoptotic signaling, while miR-144 and miR-21 influence endothelial cell survival and pro-inflammatory pathways. Members of the miR-29 family are known to affect extracellular matrix organization and trophoblast differentiation, processes that are essential for placental vascular adaptation. Collectively, these changes amplify the maternal systemic response to fetal microchimerism and contribute to the progression of preeclampsia by promoting vascular dysfunction and inflammatory activation. Evidence for these associations and mechanistic links has been described in recent studies [112,146].

Dysregulation of these miRNAs may contribute to systemic immune activation and endothelial dysfunction in preeclampsia.

Maternal effects of placenta-derived miRNAs in fetal microchimerism and preeclampsia are presented in Table 2.

**Table 2:** miRNAs associated with fetal microchimerism in preeclampsia.

miRNA	Biological Source	Interaction with Fetal Microchimerism	Mechanism of Action	Role in Preeclampsia	References
miR-517a/b/c, miR-517-5p,	Trophoblast exosomes	Co-transported with fetal microchimeric cells into maternal circulation	Modulates maternal T cells, NK cells, and monocytes; regulates immune tolerance	Impaired trophoblast invasion, incomplete spiral artery remodeling, amplified maternal inflammation	[112,114,142]
miR-519d, miR-520 family	(C19MC cluster)				
miR-210	Trophoblast EVs under hypoxia	Released alongside fetal cells into maternal blood	Increases pro-inflammatory cytokines; affects endothelial cells	Enhances systemic maternal immune activation, endothelial dysfunction	[145,147]
miR-155	Trophoblast-derived EVs	Alters maternal immune response to fetal microchimeric cells	Activates NF-κB signaling in maternal leukocytes	Promotes maternal systemic inflammation and vascular injury	[146]
miR-223	Placental exosomes	Delivered with fetal cells	Regulates angiogenesis and immune adaptation	Downregulation → maternal tissues react to fetal cells → hypertension, endothelial dysfunction	[145,147]
miR-148a/miR-152	Trophoblast exosomes	Modulates maternal recognition of fetal microchimeric cells	Regulates HLA-G expression on trophoblasts	Reduced tolerance to fetal cells → immune activation	[112]
miR-370-3p	Trophoblast EVs	Co-circulates with fetal microchimeric cells	Modulates endothelial and immune cell function	Worsens endothelial dysfunction and maternal inflammatory response	[145,147]
miR-144	Placental exosomes	Interacts with maternal immune cells	Alters macrophage and NK cell activity	Promotes pro-inflammatory state in maternal circulation	[146]
miR-21	Trophoblast exosomes	Associated with fetal cell trafficking	Modulates Treg/Th17 balance	Enhances systemic inflammation, contributes to preeclampsia	[112]
miR-29 family	Placental EVs	Delivered alongside fetal microchimeric cells	Impairs endothelial function and trophoblast differentiation	Contributes to placental hypoxia, vascular dysfunction	[112]

## 5. Immune Dysregulation in Preeclampsia

### 5.1. Maternal–Fetal Immune Interactions and Th1/Th2/Th17/Treg Balance in Normal Pregnancy

#### 5.1.1. Th1/Th2/Th17 Axis

Normal pregnancy represents a unique immunological state in which the maternal immune system tolerates the semi-allogeneic fetus while maintaining defense against pathogens. This finely tuned immune environment involves both innate and adaptive immunity, including decidual uNK cells, macrophages, dendritic cells, and T lymphocyte subsets [149,150,151]. Proper immune balance is essential to support trophoblast invasion, spiral artery remodeling, and placental development, and to prevent excessive maternal inflammation.

T cells play a fundamental role in regulating and activating immune responses. Traditionally, Th cells have been divided into Th1 and Th2 subsets.

Th1 cells produce IFN-γ and TNF-α, which are pro-inflammatory. Controlled Th1 responses are necessary for immune defense against infections and for initiating aspects of trophoblast-mediated vascular remodeling. During early pregnancy, Th1 immune responses are upregulated, supporting initial trophoblast invasion and remodeling of the uterine spiral arteries. Following placentation, the immune balance shifts toward a Th2-dominant profile, which promotes an anti-inflammatory environment that is maintained until parturition. In normal pregnancy, Th1 activity is restrained by Th2 cells and Tregs, preventing excessive inflammation [33,151,152].

Th2 cells secrete anti-inflammatory cytokines such as IL-4, IL-10, and IL-13. These cytokines promote fetal tolerance by suppressing cytotoxic maternal responses (e.g., from Th1 cells and natural killer (NK) cells) and reducing inflammation at the maternal–fetal interface. Th2 dominance indirectly supports angiogenesis and placental development through paracrine signaling to trophoblasts [32,33,151]. Earlier theories suggested that successful pregnancy depends on a shift toward Th2-dominant immunity, which suppresses Th1 responses through IL-4 production and suppresses Th17 activation through IL-10 secretion, preventing maternal immune attack against the semi-allogeneic fetus.

However, later studies showed that the Th1/Th2 paradigm alone cannot fully explain maternal–fetal immune tolerance, as both Th1 and Th2 patterns have been observed in pregnancy complications such as recurrent miscarriage and preeclampsia.

The concept has expanded to include additional T-cell subsets, particularly Th17 cells and Tregs.

The Th1/Th2/Th17/Treg system constitutes the key adaptive immune arm that regulates maternal–fetal tolerance and immune defense [33,151].

Th17 cells secrete IL-17A, promoting recruitment of neutrophils and other innate immune cells. Controlled Th17 activity protects the placenta and fetus from pathogens without causing harmful inflammation. Th17 responses are normally restrained by Tregs; imbalance toward Th17 dominance may predispose to pregnancy complications [33,151,153].

Tregs play a key role in immune tolerance by suppressing the activation and proliferation of various immune cells, including CD4+ and CD8+ T cells, B cells, NK cells, and dendritic cells. Tregs are produced centrally in the thymus and are characterized by high forkhead box protein P3 (FoxP3) expression and function to suppress excessive maternal immune activation [154]. They maintain tolerance toward fetal antigens by inhibiting Th1 and Th17 pro-inflammatory cells and prevent immune-mediated placental damage. Tregs also modulate uNK cell activity and cytokine secretion, contributing to balanced immunity and tissue remodeling [150,151,153].

Through these mechanisms, Tregs help maintain immune homeostasis and protect the fetus from maternal immune rejection.

#### 5.1.2. uNK Cell Interactions

Decidual uNK are predominant lymphocytes in the first trimester and interact with trophoblast HLA molecules, secreting VEGF, PlGF, IFN-γ, and IL-8. These secretions guide spiral artery remodeling and promote EVTs invasion.

Proper Th1/Th2/Th17/Treg balance influences decidual uNK phenotype and cytokine output, ensuring vascular adaptation and maternal–fetal tolerance [15,152,153].

The coordinated activity of Th1/Th2/Th17/Treg cells, together with decidual uNK cells, ensures maternal immune tolerance, proper placental development, and defense against pathogens.

Disruption of this balance, such as Th1/Th17 dominance or Treg deficiency, can impair trophoblast invasion, reduce spiral artery remodeling, and predispose to pregnancy disorders including preeclampsia [15,32,151,155].

- Activation of the maternal immune system in preeclampsia

While abnormal placentation has long been recognized as a key event, immune dysregulation at the maternal–fetal interface is increasingly recognized as central to preeclampsia pathogenesis, linking defective trophoblast invasion, shallow spiral artery remodeling, and systemic maternal inflammation.

Hyperactivation of maternal immune cells—including monocytes, neutrophils, NK cells, and T lymphocytes—leads to elevated levels of pro-inflammatory cytokines TNF-α, IL-6, IL-17A, and IFN-γ, which impair trophoblast invasion, spiral artery remodeling, and endothelial function [34,155,156,157,158,159,160,161]. miRNAs, particularly miR-155 and miR-210, further amplify immune-mediated inflammation and angiogenic dysfunction [160,161].

During normal pregnancy, the maternal immune system is carefully modulated to tolerate semi-allogeneic fetal antigens while maintaining pathogen defense. In preeclampsia, this tolerance is disrupted, and there is hyperactivation of innate and adaptive immune cells, including monocytes, neutrophils, dendritic cells, and pro-inflammatory T lymphocytes [33,81,155,158,162].

This hyperactivation results in elevated levels of pro-inflammatory cytokines, TNF-α, IL-6, IL-17A, and IFN-γ, with multiple downstream effects.

- TNF-α (Tumor Necrosis Factor-alpha)

TNF-α is secreted by activated macrophages, uNK cells, and Th1 lymphocytes. In preeclampsia it has been linked to endothelial dysfunction, placental oxidative stress, and impaired trophoblast invasion. Thus, TNF-α reduces NO bioavailability and upregulates adhesion molecules (vascular cell adhesion molecule-1 = VCAM-1, intercellular adhesion molecule-1 = ICAM-1), promoting leukocyte adhesion and vascular inflammation [155,157,158]. Also, TNF-α stimulates ROS production in trophoblasts and endothelial cells, leading to lipid peroxidation and apoptosis [32,158]. TNF-α limits extravillous trophoblast migration and spiral artery remodeling by inhibiting matrix metalloproteinases (MMP-2, MMP-9) [32,155,156]. Moreover, TNF-α induces IL-6 and IL-17A secretion, amplifying local and systemic inflammation [158,159]. Elevated maternal TNF-α levels correlate with severity of hypertension and proteinuria [155,158].

- IL-6 (Interleukin-6)

IL-6 is produced by trophoblasts, monocytes, and stromal cells. In preeclampsia IL-6 promotes Th1 differentiation and inhibits Treg development, reinforcing a pro-inflammatory profile [157,159]. Also, it upregulates VEGF antagonists (sFlt-1) and increases vascular permeability, contributing to maternal hypertension [155,157]. IL-6 stimulates hepatocytes to produce CRP, connecting placental inflammation with systemic maternal inflammation [158,159]. It alters proliferation and cytokine secretion, reducing vascular remodeling capacity [153,155].

- IL-17A (Interleukin-17A)

IL-17A is secreted mainly by Th17 cells. In preeclampsia it has a role in neutrophil recruitment, enhancing infiltration into the placenta, exacerbating oxidative stress and tissue damage [155,156,159]. By stimulating TNF-α and IL-6 production, it creates a positive feedback loop that intensifies inflammation [155,159]. Increasing vascular permeability and ROS production, endothelial dysfunction appear, thus contributing to maternal hypertension [157,158]. Enhancing uNK cytotoxicity and reducing Treg-mediated tolerance occurred, impairing spiral artery remodeling [155,157,160].

- IFN-γ (Interferon-gamma)

IFN-γ is secreted by Th1 cells and NK cells. In preeclampsia, it determines trophoblast inhibition by limiting EVT invasion and MMP expression, resulting in shallow spiral artery remodeling [155,156,158]. Also, it promotes M1 macrophage polarization, increasing local cytokine production and oxidative stress which determine placental inflammation [155,157]. IFN-γ acts synergistically with TNF-α to upregulate adhesion molecules and induce apoptosis in vascular endothelial cells [158,159]. One of the miRNA which interacts with IFN-γ is miRNA-155. This miRNA amplifies IFN-γ-mediated pro-inflammatory signaling, destabilizing maternal–fetal tolerance [160,161].

- Role of uterine natural killer (uNK) cells

uNK cells are predominant in the decidua during the first trimester, critical for spiral artery remodeling by secreting angiogenic factors (VEGF, PlGF) and cytokines (IFN-γ, IL-8) that promote EVT migration and invasion [153,155,159]. In preeclampsia, altered receptor expression and cytotoxic KIR/HLA mismatches impair trophoblast recognition [153,159]. Reduced angiogenic cytokine secretion limits vascular adaptation, contributing to placental hypoxia and systemic endothelial stress [152,153,159]. miRNAs, particularly miR-155, shift uNK profiles toward a pro-inflammatory phenotype, further compromising vascular remodeling [160,161].

### 5.2. Maternal–Fetal Immune Interactions and Th1/Th2/Th17/Treg Imbalance in Preeclampsia

Physiologic pregnancy shifts toward Th2 and Treg dominance to maintain fetal tolerance. In preeclampsia, elevated IFN-γ and TNF-α amplify inflammation and endothelial injury [34,81,156]. Reduced FoxP3 + Tregs fail to suppress maternal immune activation [157,158,162]. Enhanced IL-17A secretion promotes neutrophil recruitment, vascular inflammation, and oxidative stress [157,158]. Placental miRNAs such as miR-155 reinforce this immune imbalance by skewing T-cell differentiation toward Th1/Th17 and reducing Treg stability [160,161].

Preeclamptic placentas exhibit chronic inflammation, with infiltrates of activated macrophages (M1), cytotoxic NK cells, and pro-inflammatory T/B lymphocytes [81,155,162]. Consequences include chemokine dysregulation, endothelial injury and impaired angiogenesis. Elevated monocyte chemoattractant protein-1 (CCL2) and C–X–C motif chemokine ligand 10 (CXCL10) recruit immune cells, amplifying inflammation [156,157]. Cytokines and miRNA-loaded exosomes enter maternal circulation, inducing systemic endothelial dysfunction [158,160]. Inflammatory mediators disrupt VEGF/PlGF signaling, impairing vascular growth and remodeling [153,155].

These processes create a feed-forward loop of placental inflammation, immune dysregulation, and maternal vascular dysfunction.

**Figure 5:**
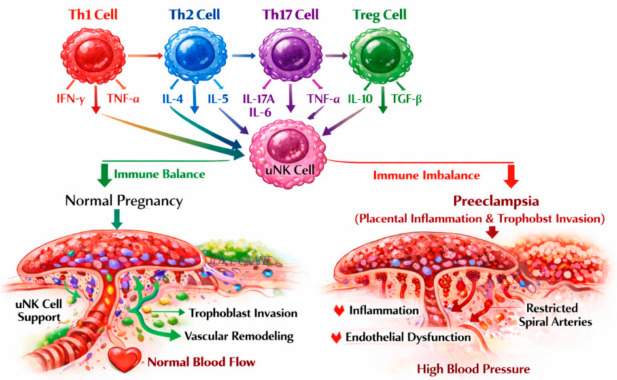
Th1/Th2/Th17/Treg immune balance at the maternal–fetal interface in normal pregnancy and preeclampsia. The diagram illustrates the interactions between T helper cell subsets (Th1, Th2, Th17, and Treg), their associated cytokines (IFN-γ, TNF-α, IL-4, IL-5, IL-17A, IL-6, IL-10, and TGF-β), and uNK cells in regulating placental development. In normal pregnancy, a predominance of Th2 and Treg responses promotes immune tolerance, supports uNK-mediated trophoblast invasion, and enables proper spiral artery remodeling and placental perfusion. In preeclampsia, a shift toward Th1/Th17-mediated inflammation disrupts immune regulation, leading to impaired trophoblast invasion, inadequate spiral artery remodeling, placental inflammation, and endothelial dysfunction.

The role of the Th1/Th2/Th17 axis in normal pregnancy and preeclampsia is presented in Figure 5.

Taken together, these findings are further supported by recent high-resolution studies that provide a more nuanced understanding of immune alterations in preeclampsia.

Overall, recent advances using single-cell and single-nucleus transcriptomic approaches further refine the understanding of preeclampsia as a cell type-specific and spatially organized disorder of the placenta, rather than a uniform pathological process. These studies demonstrate that distinct placental cell populations exhibit divergent transcriptional programs, reflecting both adaptive and maladaptive responses to placental stress. A key finding is the presence of pro-inflammatory and stress-associated signatures across multiple cell types, including trophoblasts, immune cells, and stromal populations, particularly involving pathways related to hypoxia, oxidative stress, interferon signaling, and immune activation. This supports the concept that placental dysfunction in preeclampsia is tightly linked to a sustained inflammatory microenvironment.

Importantly, significant alterations in trophoblast differentiation and function have been identified, including impaired invasive phenotypes and disrupted signaling pathways involved in vascular remodeling, providing mechanistic support for defective spiral artery remodeling. In parallel, these studies reveal profound immune remodeling at the maternal–fetal interface, characterized by shifts in macrophage polarization, altered NK cell activity, and dysregulated T-cell responses. These findings reinforce the concept that immune imbalance in preeclampsia is not merely an exaggerated inflammatory response, but rather a complex reprogramming of immune cell function, accompanied by disrupted cell–cell communication networks, particularly involving ligand–receptor interactions between trophoblasts and immune cells [163].

In this context, accumulating evidence highlights that immune alterations in preeclampsia are complex, dynamic, and not limited to a simple state of immune activation. Instead, the disease is characterized by a dual pattern of immune dysregulation, involving the simultaneous activation of specific inflammatory cell subsets—particularly monocytes—and suppression of key adaptive immune functions. The observed expansion of Treg cells alongside reduced cytotoxic activity and impaired lymphocyte activation suggests that, despite a pro-inflammatory milieu, protective and regulatory immune responses are not effectively coordinated, contributing to inadequate immune tolerance and defective placental development.

Furthermore, monocyte subsets play a central role in driving systemic inflammation, with enhanced pathways related to interferon signaling, complement activation, angiogenesis, and coagulation, all of which are consistent with known features of preeclampsia. These findings also support the concept that immune dysregulation is cell type-specific and highly heterogeneous, as demonstrated by single-cell transcriptomic analyses, which provide deeper insight into immune cell functional diversity beyond conventional bulk approaches [164].

Complementary multi-omics studies integrating peripheral transcriptomic data with placental single-cell landscapes further provide a comprehensive, multi-layered view of preeclampsia. These analyses confirm that the disease involves complex immune dysregulation across both innate and adaptive pathways, with a prominent contribution of neutrophil-driven inflammation and broader immune–metabolic disturbances. The identification of key candidate genes such as T-cell Leukemia/Lymphoma 1A (TCL1A), C-type Lectin Domain Family 2 Member B (CLEC2B), and Lectin, Galactoside-Binding, Soluble, 9 (LGALS9) across multiple analytical platforms underscores their central role in preeclampsia pathogenesis. Their cell type-specific expression patterns demonstrate that distinct immune populations within the placenta—including B cells, NK cells, monocytes, and Hofbauer cells—contribute differentially to disease development, thereby linking systemic immune alterations with local placental dysfunction [165].

Moreover, the convergence of peripheral blood transcriptomic signals with placental single-cell data supports a critical concept: maternal circulating biomarkers can reflect early placental pathology, offering potential for non-invasive early detection and risk stratification. At the same time, these findings emphasize that preeclampsia is a biologically heterogeneous disorder, with variability influenced by different maternal risk factors and clinical subtypes, particularly early-onset disease, highlighting the need for larger and more stratified studies.

The integration of single-cell and multi-omics approaches provides novel insight into the immunopathogenesis of preeclampsia and identifies clinically relevant molecular targets. The combination of these approaches with machine learning further highlights the potential for cell type-specific biomarkers and diagnostic models, supporting the development of early diagnostic tools and targeted therapeutic interventions for improved management of this complex pregnancy disorder.

## 6. Preventive Therapeutic Approaches Targeting Preeclampsia Pathogenesis

Several pharmacological strategies with anti-inflammatory and immunomodulatory properties are currently used or investigated for the prevention and management of preeclampsia [166]. The most evidence-based preventive therapy is low-dose aspirin (81–150 mg daily), usually initiated before 16 weeks of gestation. Aspirin inhibits thromboxane A2 production, improving trophoblast invasion and spiral artery remodeling while reducing complement activation in the placenta. This intervention is recommended for women with significant risk factors for preeclampsia and has been shown to decrease the incidence of preterm preeclampsia [167,168,169].

Heparin, often administered together with aspirin in women with antiphospholipid antibody syndrome, also contributes to improved uteroplacental blood flow and may modulate immune dysregulation by reducing trophoblast C5a production [170,171,172].

Additionally, hydroxychloroquine is recommended during pregnancy for patients with systemic lupus erythematosus, as it lowers the risk of disease flares and may reduce preeclampsia risk. This antimalarial drug exerts anti-inflammatory, antioxidant, and antithrombotic effects, while also limiting complement-mediated immune activation and T-cell stimulation [173].

IL-17 is an important mediator of inflammation and antibacterial immunity and has been associated with impaired immune tolerance in preeclampsia as well as in autoimmune and inflammatory disorders. Therapeutic agents targeting IL-17, such as the monoclonal antibody secukinumab [167,168,169,174,175,176,177], are already used in diseases like psoriasis and lupus. Dual inhibitors targeting IL-17 and B-cell activating factor (BAFF), such as tibulizumab, are also being explored to reduce inflammatory responses. Additionally, biological therapies that block TNF-α have shown efficacy in inflammatory diseases and have been used safely during pregnancy, suggesting potential value in correcting the Th1-driven inflammatory imbalance observed in preeclampsia [167,168,169,174,175,176,177,178,179].

Complement-mediated inflammation and adaptive T-cell polarization are key features of preeclampsia, resembling mechanisms observed in autoimmunity and transplant rejection. Because preeclampsia is associated with impaired Treg function, therapeutic strategies aimed at enhancing Treg activity may represent potential preventive or therapeutic approaches. Proposed strategies include improving preconception immune priming to paternal antigens, promoting endogenous Treg expansion through lifestyle or pharmacological interventions, and developing autologous Treg cell therapies, which are currently being investigated in several autoimmune and transplant-related clinical trials [174,175,176,177].

## 7. Clinical Implications and Future Perspectives

Identification of women at high risk of preeclampsia is essential for prevention and early intervention. Preventive strategies include low-dose aspirin therapy, close antenatal monitoring, and biomarker screening. The use of angiogenic biomarkers such as the sFlt-1/PlGF ratio has improved diagnostic accuracy.

Because placental miRNAs are released into maternal circulation through exosomes and extracellular vesicles, they represent promising non-invasive biomarkers for early prediction of preeclampsia [160,161]. Circulating miRNAs frequently elevated in preeclampsia include miR-210, miR-155, miR-16, and miR-29b. Detection of these miRNAs in maternal plasma during early pregnancy may allow risk stratification and earlier clinical monitoring [180]. Recent studies using next-generation sequencing and circulating exosomal miRNA profiling have identified specific miRNA signatures that may predict preeclampsia before clinical onset [105].

Moreover, preeclampsia rises from maternal immune hyperactivation, dysregulated uNK activity, Th1/Th2/Th17/Treg imbalance, and chronic placental inflammation, amplified by circulating miRNAs such as miR-155. This immune–cytokine network leads to shallow trophoblast invasion, oxidative stress, and systemic vascular pathology. Immunomodulatory strategies aimed at restoring Treg cell function, uNK cell activity, and miRNA regulatory balance may help improve placental development and contribute to better maternal and pregnancy outcomes [34,81].

Future studies should focus on novel biomarkers for early prediction, the role of extracellular vesicles and exosomes, microbiome influences on pregnancy complications, and personalized medicine approaches.

### Novel Contributions of This Review

While future perspectives highlight potential research directions, the present review contributes a distinct integrative perspective on preeclampsia pathogenesis.

First, it shifts from a predominantly linear conceptual model toward a network-based framework in which placental hypoxia, immune dysregulation, and epigenetic mechanisms continuously interact and amplify each other. This bidirectional interplay is emphasized more strongly than in conventional descriptions.

Second, the review introduces a clearer translational dimension by systematically linking mechanistic pathways to clinically relevant biomarkers and therapeutic strategies, including circulating microRNAs, immune modulation, and Treg-targeted approaches, supporting the transition toward personalized medicine.

Third, it highlights the role of specific microRNA networks (such as miR-210, miR-155, and C19MC cluster members) as central integrators of hypoxia, impaired trophoblast invasion, angiogenic imbalance, and endothelial dysfunction. In parallel, fetal microchimerism is presented as an underexplored mechanism that may amplify maternal systemic inflammation.

Finally, the review reframes preeclampsia as a multisystem immuno-vascular disorder with long-term maternal consequences, integrating cardiovascular risk, immune memory, and persistent endothelial dysfunction into a unified pathogenic continuum.

Overall, the novelty of this work lies not in proposing entirely new mechanisms, but in providing a multi-level, integrative, and clinically oriented synthesis of how established pathways interact.

## 8. Conclusions

Preeclampsia is a complex disorder resulting from the interaction between genetic, metabolic, immunological, and environmental factors. Abnormal placentation and angiogenic imbalance initiate a cascade of events leading to systemic endothelial dysfunction and the clinical manifestations of the disease. Maternal risk factors such as obesity, diabetes, chronic hypertension, and advanced maternal age contribute to disease development through shared mechanisms, including systemic inflammation, oxidative stress, endothelial dysfunction, and angiogenic imbalance.

MicroRNAs play a fundamental role in the pathophysiology of preeclampsia by regulating key biological processes including angiogenesis, trophoblast invasion, inflammation, and endothelial function. Among these regulators, miR-210 and miR-155 have emerged as critical mediators linking placental hypoxia, immune activation, and vascular dysfunction. Microchimerism has been implicated in the pathophysiology of preeclampsia. According to the two-stage model of the disease, fetal microchimerism through C19MC-miRNAs, especially miR-519d and miR-517-5p, affects trophoblast function and may function as a mechanistic link connecting placental dysfunction in early pregnancy with the subsequent development of maternal cardiovascular complications.

In addition to their mechanistic involvement in disease development, circulating miRNAs represent promising non-invasive biomarkers for early detection and risk prediction of preeclampsia. Future research focusing on miRNA-based diagnostic tools and targeted therapies may significantly improve clinical management and maternal–fetal outcomes in pregnancies complicated by preeclampsia.

The Th1/Th2/Treg balance plays a central role in the pathogenesis of preeclampsia. A shift toward Th1 dominance and reduced Treg activity promotes a pro-inflammatory environment at the maternal–fetal interface, impairing trophoblast invasion, spiral artery remodeling, and placental vascular development. This immune imbalance contributes to systemic endothelial dysfunction, oxidative stress, and the clinical manifestations of preeclampsia. Therapeutic strategies that restore Treg function or rebalance Th1/Th2 responses hold promise for mitigating disease severity and improving maternal and fetal outcomes.

Improved understanding of these mechanisms will facilitate earlier diagnosis, improving prevention strategies, better risk stratification, and the development of targeted therapies aimed at reducing the global burden of preeclampsia.

## Data Availability

No new data were created or analyzed in this study. Data sharing is not applicable to this article.

## Abbreviations

The following abbreviations are used in this manuscript:

ACE	angiotensin-converting enzyme
AGT	angiotensinogen gene
AGTR1	angiotensin II type 1 receptor gene
AT1-Aas	angiotensin II type 1 receptors
BAFF	B-cell activating factor
BMI	body mass index
C19MC	chromosome 19 microRNA cluster
CCL2	monocyte chemoattractant protein-1
CHD	coronary heart disease
CLEC2B	C-type Lectin Domain Family 2 Member B
CRP	C-reactive protein
CXCL10	C–X–C motif chemokine ligand 10
EFNA3	ephrin A3
eNOS	endothelial nitric oxide synthase
ERAP1/ERAP2	endoplasmic reticulum aminopeptidase 1/endoplasmic reticulum aminopeptidase 2
EVTs	extravillous trophoblasts
FoxP3	forkhead box protein P3
GWAS	genome-wide association studies
HIF-1α	hypoxia-inducible factor-1α
HLA	human leukocyte antigen
HOXA9	homeobox A9
ICAM-1	intercellular adhesion molecule-1
IFN-γ	interferon gamma
IL-4/IL-6/IL-10/IL-13/IL-17/IL-1β	interleukin-4/interleukin-6/interleukin-10/interleukin-13/interleukin-17/interleukin-1β
ISCU	iron-sulfur cluster scaffold protein
ISSHP	international society for the study of hypertension in pregnancy
KCMF1	potassium channel modulatory factor 1
KIR	killer cell immunoglobulin-like receptor
LGALS9	Lectin, Galactoside-Binding, Soluble, 9
MHC	major histocompatibility complex
MI	myocardial infarction
miRNAs	microRNAs
mRNA	messenger RNA
NO	nitric oxide
NK	natural killer
NF-κB	nuclear factor kappa B
PIGF	placental growth factor
RAAS	renin–angiotensin–aldosterone system
ROS	reactive oxygen species
sEng	soluble endoglin
sFlt-1	soluble fms-like tyrosine kinase-1
STOX1	storkhead box 1
TCL1A	T-cell Leukemia/Lymphoma 1A
Th1/Th2/Th17	T helper 1/T helper 2/T helper 17 cells
TNF-α	tumor necrosis factor-α
Tregs	regulatory T cells
uNK	uterine natural killer
VCAM-1	vascular cell adhesion molecule-1
VEGF	vascular endothelial growth factor
